# Household Exposure to Pesticides and Risk of Childhood Hematopoietic Malignancies: The ESCALE Study (SFCE)

**DOI:** 10.1289/ehp.10596

**Published:** 2007-09-25

**Authors:** Jérémie Rudant, Florence Menegaux, Guy Leverger, André Baruchel, Brigitte Nelken, Yves Bertrand, Catherine Patte, Hélène Pacquement, Cécile Vérité, Alain Robert, Gérard Michel, Geneviève Margueritte, Virginie Gandemer, Denis Hémon, Jacqueline Clavel

**Affiliations:** 1 INSERM (Institut national de la santé et de la recherche médicale), Villejuif, France; 2 Univ Paris-Sud, Villejuif, France; 3 AP HP (Assistance Publique des Hôpitaux de Paris), Hôpital Armand Trousseau, Paris, France; 4 AP HP (Assistance Publique des Hôpitaux de Paris), Hôpital Saint-Louis and Hôpital Robert-Debré, Paris, France; 5 Hôpital Jeanne de Flandre, Lille, France; 6 Hôpital Debrousse, Lyon, France; 7 Institut Gustave Roussy, Villejuif, France; 8 Institut Curie, Paris, France; 9 Hôpital Pellegrin Tripode, Bordeaux, France; 10 Hôpital des Enfants, Toulouse, France; 11 Hôpital La Timone, Marseille, France; 12 Hôpital Arnaud de Villeneuve, Montpellier, France; 13 CHU-hôpital Sud, Rennes, France; 14 French National Registry of Childhood Blood malignancies (RNHE), Villejuif, France

**Keywords:** acute leukemia, children, Hodgkin lymphoma, non-Hodgkin lymphoma, pesticide, pregnancy

## Abstract

**Objectives:**

We investigated the role of household exposure to pesticides in the etiology of childhood hematopoietic malignancies.

**Methods:**

The national registry-based case–control study ESCALE (Etude sur les cancers de l’enfant) was carried out in France over the period 2003–2004. Population controls were frequency matched with the cases on age and sex. Maternal household use of pesticides during pregnancy and paternal use during pregnancy or childhood were reported by the mothers in a structured telephone questionnaire. Insecticides (used at home, on pets, or for garden crops), herbicides, and fungicides were distinguished. We estimated odds ratios (ORs) using unconditional regression models closely adjusting for age, sex, degree of urbanization, and type of housing (flat or house).

**Results:**

We included a total of 764 cases of acute leukemia (AL), 130 of Hodgkin lymphoma (HL), 166 of non-Hodgkin lymphoma (NHL), and 1,681 controls. Insecticide use during pregnancy was significantly associated with childhood AL [OR = 2.1; 95% confidence interval (CI), 1.7–2.5], both lymphoblastic and myeloblastic, NHL (OR = 1.8; 95% CI, 1.3–2.6), mainly for Burkitt lymphoma (OR = 2.7; 95% CI, 1.6–4.5), and mixed-cell HL (OR = 4.1; 95% CI, 1.4–11.8), but not nodular sclerosis HL (OR = 1.1; 95% CI, 0.6–1.9). Paternal household use of pesticides was also related to AL (OR = 1.5; 95% CI, 1.2–1.8) and NHL (OR = 1.7; 95% CI, 1.2–2.6); but for AL the relationships did not remain after adjustment for maternal pesticide use during pregnancy.

**Conclusion:**

The study findings strengthen the hypothesis that domestic use of pesticides may play a role in the etiology of childhood hematopoietic malignancies. The consistency of the findings with those of previous studies on AL raises the question of the advisability of preventing pesticide use by pregnant women.

Hematopoietic malignancies are the most common childhood cancers, with world age-standardized incidence rates of 43.1, 6.7, and 8.9 per million children in France for leukemia, Hodgkin lymphoma (HL), and non-Hodgkin lymphoma (NHL), respectively (Clavel et al. 2004). The etiology of those malignancies remains largely unknown. Some epidemiologic studies have suggested that pesticides might increase the risk of childhood hematopoietic malignancies (Daniels et al. 1997; Infante-Rivard and Scott Weichenthal 2007; Jurewicz and Hanke 2006; Nasterlack 2006, 2007; Zahm and Ward 1998). Furthermore, the International Agency for Research on Cancer (IARC) has classified the occupational spraying of insecticides as probably carcinogenic to humans (group 2A); adult lymphoma is one of the main cancers suspected (IARC 1991). Children can be exposed to pesticides *in utero* or during childhood through their parents’ work, domestic use, or the general environment (residues in food, water, air, and soil). It is not clear which sources of pesticide exposure are the most important for children, and household pesticide exposure may be a major exposure for children (Bradman and Whyatt 2005; Grossman 1995). No French survey on household pesticide use is available, but surveys conducted in North America and the United Kingdom reported high rates of household use or storage of pesticides (Adgate et al. 2000; Grey et al. 2006).

This study investigated the relationship between household exposure to pesticides and the risks of childhood acute leukemia (AL), HL, and non-NHL, focusing on intrauterine exposures, using data generated by the French national population-based case–control study, ESCALE (Etude sur les cancers de l’enfant).

## Patients and Methods

We conducted the ESCALE study in 2003 and 2004 to investigate the role of infectious, environmental, and genetic factors in four childhood neoplastic diseases (leukemia, lymphoma, neuroblastoma, and brain tumor).

### Cases and controls ascertainment

#### Cases

The cases were identified directly by the investigators assigned to each French pediatric oncology hospital department, with the support of the French National Registry of Childhood Blood Malignancies (Clavel et al. 2004). For the cases to be eligible, leukemia or lymphoma was to have been newly diagnosed between 1 January 2003 and 31 December 2004. The cases were also required to be < 15 years of age and resident in France at the time of diagnosis. Cases who had been adopted, whose biological mother had died, whose mother did not speak French, or whose mother presented with a psychiatric disorder were not eligible. For ethical reasons, the children who had died or who were receiving hospital palliative care were not eligible. Of the 1,316 cases (938 AL, 171 HL, 207 NHL) of childhood hematopoietic malignancies identified during the study period, 1,182 (843 AL, 152 HL, 186 NHL) cases were eligible. The reasons for noneligibility were the child’s death (34 AL, 3 HL, 7 NHL), hospital palliative care (7 AL, 1 HL, 1 NHL), biological mother’s death (10 AL, 3 NHL), non-French-speaking mother (29 AL, 11 HL, 8 NHL), or mother with serious psychiatric disorders (15 AL, 4 HL, 2 NHL).

The participation rates were 91, 86, and 88% for acute leukemia, HL, and NHL, respectively.

#### Controls

The controls were randomly selected from the French population using a quota sampling method. A first sample of 60,000 addresses representative of the French population in terms of the 22 administrative regions and nine degrees of urbanization was randomly extracted from the French national telephone directory (plus randomly generated unlisted numbers). Then quotas were applied. The quotas were designed to make the controls similar to all the cases of all types of cancer in terms of age and sex, using the French National Registry of Childhood Blood Malignancies (Clavel et al. 2004) and the Regional Childhood Cancer Registries (Desandes et al. 2004) as references. The following age strata were used for quota sampling: 0–1 years, 2 years, 3 years, 4 years, 5–6 years, 7–8 years, 9–11 years, 12–14 years. Additional quotas were used to ensure that the control group was also representative of the French general population in terms of the number of children < 15 years of age living in the household, based on the 1999 population census [Institut national de la statistique et des études économiques (INSEE) 1999]. Like the cases, the controls were not to have been adopted and were to have a biological mother who could be interviewed (alive, not presenting with a serious psychiatric disorder, and French speaking). Of the 50,217 phone numbers dialed, 22,584 did not connect to a home number, 24,410 were ineligible, and for 862, the respondent hung up before eligibility could be checked. For the 2,361 remaining numbers, there were 679 refusals to participate. Thus, 1,682 mothers were interviewed (71.2%). We excluded one control with a history of neuroblastoma and thus finally included 1,681 children as controls.

### Data collection

The same trained interviewers carried out the telephone interviews with the cases’ and controls’ biological mothers, using structured questionnaires. The cases’ mothers were interviewed an average of 6 months after the diagnosis. The telephone questionnaire elicited information on demographic and socioeconomic characteristics, childhood environment, lifestyle, and personal and familial medical history. The questions relating to pesticide exposure included household use of pesticides during pregnancy by the mother and during pregnancy or childhood by the father. Insecticides used at home, on pets, or for garden crops; herbicides used as “weed killers”; and fungicides were distinguished. The choices proposed were “ever used,” “never used,” or “not known.” We also asked the mothers whether they had been exposed to pesticides at work during pregnancy and whether they had had an agricultural occupation during pregnancy. The questionnaire also detailed residential history since conception. For each move, the type of housing (flat, house, or farm) and the name of the town and postal/area code were elicited to identify the degree of urbanization, on the basis of the 1999 census data (INSEE 1999).

### Statistical analysis

We carried out separate analyses by childhood hematopoietic malignancy (i.e., AL, HL, and NHL). We estimated the odds ratios (ORs) and their 95% confidence intervals (CIs) using unconditional logistic regression, with adjustment for the stratification variables age and sex. The analyses were restricted to cases and controls ≥ 5 years of age for HL and ≥ 2 years of age for NHL because of the small numbers of lymphoma cases in the lowest age groups. We analyzed separately household maternal pesticide use during pregnancy and paternal use since conception. For each kind of pesticide use tested (any pesticide, any insecticide, home insecticides, pet insecticides, garden crop insecticides, herbicides, fungicides, maternal and paternal agricultural occupation, maternal occupational contact with pesticide during pregnancy), the reference group was the group of children whose mother’s response to the questionnaire was “never used” for the corresponding use of pesticide and the parent considered. We initially considered “not known” answers as missing data, but we also performed further sensitivity analyses considering the “not known” answers to be “never used,” “ever used,” and “ever used” for controls and “never used” for cases.

We adjusted for potential confounders including even factors that were associated not significantly with pesticide use and with any childhood hematopoietic malignancy—AL, HL, or NHL. Thus, systematic adjustment for the degree of urbanization of the place of residence and for the type of housing was conducted. All the analyses on AL were adjusted for birth order, which was significantly associated with both AL and pesticide use. In addition, although parental socio-professional category and degree of education were not related to pesticide use, the analyses were repeated with adjustment for those variables. Similarly, adjustments were made for family history of cancer, early infections, breast-feeding and child care attendance, which had previously been shown to be related to childhood leukemia or lymphoma in the literature and in the ESCALE study (Rudant et al. 2007).

To evaluate possible mutual confounding between different pesticide exposures, maternal and paternal pesticide uses were included in the same models, and the two variables were also combined as follows: no pesticide use by either parent, maternal pesticide use only, paternal pesticide use only, pesticide use by both parents. Finally, the maternal and paternal uses of insecticide, herbicide, and fungicide were combined.

Additional analyses by AL subtype [acute lymphoblastic leukemia (ALL) and acute myeloblastic leukemia (AML)], ALL subtype (common B-cell ALL, mature B-cell ALL, and T-cell ALL), HL subtype (nodular sclerosis, mixed cellularity and lymphocyte predominant), and NHL subtype (Burkitt, B-cell lymphoblastic, T-cell lymphoblastic, and anaplastic large cell) were carried out. We used polytomous logistic regression models for the analyses by AL subtype. Age classes were grouped for some subtype analyses when the number of children in some age strata was insufficient.

We used the SAS software package (version 9; SAS Institute Inc., Cary, NC, USA) for all the analyses. All *p*-values were two-tailed.

We have complied with all applicable requirements of the international regulations, including ethic committee (Direction Générale de la Santé 2003/0259). Participants gave oral informed consent to interview before the study (and written consent to biological sampling).

## Results

A total of 1,060 incident cases of hematopoietic malignancies were included: 764 cases of AL (646 ALL including 544 common B-cell ALL, 27 mature B-cell ALL, 67 T-cell ALL and 8 unspecified ALL, 100 AML and 18 unspecified or biphenotypic AL), 130 of HL, of whom 128 were > 4 years of age (79 nodular sclerosis, 19 mixed-cell, 18 lymphocyte predominant and 12 unspecified HL), and 166 NHL of whom 165 were > 1 year of age (76 Burkitt, 39 B-cell lymphoblastic, 27 T-cell lymphoblastic, 21 anaplasic large cell, and 2 unspecified NHL).

### Comparability of cases and controls

The distribution of the cases and controls by the quota variable combining age and sex is shown in Table 1. The cases and controls were similar with respect to that variable for the study as a whole, but not for each hematopoietic malignancy. In particular, the lymphoma cases were significantly older, on average, than the controls. All the strata contained more than one control per case for adjustment, with more controls per lymphoma case in the youngest strata. The control parents were slightly more educated and had a higher professional status than the parents of the AL and HL cases (Table 2). There was no difference between the AL and HL cases and the controls with regard to the degree of urbanization of the place of residence at the time of interview, but slightly more NHL cases than controls lived in semiurban and urban areas (Table 2). In contrast, we observed no difference between any of the case groups and controls with respect to the degree of urbanization at the time of conception (data not shown). Slightly more controls than cases appeared to have lived at least once in a house, either during the pregnancy or after birth (Table 2). We observed no difference between the cases and the controls with regard to the child’s contacts with dogs or cats.

### Exposure to pesticides

The use of pesticides at least once during pregnancy was reported by mothers of 52% of the AL cases and by mothers of 37% of the controls, by mothers of 53% of the NHL cases and by mothers of 39% of the corresponding controls > 1 year of age, and by mothers of 47% of the HL cases and by mothers of 42% of the corresponding controls > 5 years of age. Paternal use of pesticides during pregnancy or childhood was more frequent than maternal use (56% of control fathers). Maternal use of insecticides at home during pregnancy was far more frequent than herbicide use (31% and 5% of control mothers, respectively). In contrast, control fathers used insecticides and herbicides in similar numbers (43% and 41%, respectively). As expected, among controls, parental use of pesticide varied with both the degree of urbanization and the type of housing. The use of pesticides during pregnancy was declared by 42%, 39%, and 34% of control mothers living in rural, mixed urban, and urban areas, respectively, and by 48%, 42%, and 31% of control mothers living in a farm, a house, and a flat. Paternal use of pesticides was even more distinct, with 72% 61%, and 44% in rural, mixed urban, and urban areas, and 86%, 67%, and 26% when living in a farm, a house, and a flat.

The ORs associated with household parental use of pesticides are shown in Table 3. Control mothers (2.7%) answered less often than case mothers (5.1%) that they did not know whether they had ever or never used pesticides during pregnancy. Maternal household use of any pesticide during pregnancy was significantly more frequent in AL (OR = 2.2; 95% CI, 1.8–2.6) and NHL (OR = 1.9; 95% CI, 1.3–2.6) cases than in controls. This was mostly linked to home and pet insecticide use for AL and to garden crop insecticide use for NHL. Herbicide use during pregnancy was also associated with AL (OR = 1.5; 95% CI, 1.0–2.2). No association between maternal pesticide use and HL was observed regardless of pesticide type. Paternal insecticide use during pregnancy or childhood was also significantly and positively associated with AL (OR =1.4; 95% CI, 1.2–1.7) and NHL (OR = 1.5; 95% CI, 1.0–2.1), and paternal herbicide use was associated with NHL (OR = 1.5; 95% CI, 1.0–2.2).

Neither maternal occupational pesticide exposure during pregnancy nor paternal occupation as a farmer was associated with any of the hematopoietic malignancies (Table 3). On the contrary, maternal occupation as a farmer during pregnancy was negatively associated with childhood AL (OR = 0.2; 95% CI, 0.1–0.8). After exclusion of children whose parents had worked as farmers, a positive nonsignificant association was observed between maternal occupational exposure to pesticides during pregnancy and AL (OR = 1.5; 95% CI, 0.8–2.6) or NHL (OR = 1.4; 95% CI, 0.5–3.8), but not with HL (OR = 0.3; 95% CI, 0–2.6).

Table 4 shows the associations by combining the various kinds of pesticide. The use of any pesticide by the mother only, without any paternal use, was significantly more frequent for cases than for controls (AL: OR = 2.7; 95% CI, 1.9–3.8; HL: OR = 3.4; 95% CI, 1.6–7.3; NHL: OR = 3.3; 95% CI, 1.7–6.5). The same associations for insecticide use as for pesticide use were observed. The use of herbicide by the mother only was also significantly associated with AL, but very few mothers (5 controls and 4 AL cases) were involved.

In models including maternal and paternal uses of pesticides, the association between AL and paternal pesticide use was no longer observed (OR = 1.1; 95% CI, 0.9–1.4), whereas the association between AL and maternal use of pesticide remained (OR = 2.0; 95% CI, 1.6–2.4). For HL and NHL, significant negative interactions with maternal and paternal exposures precluded adjusted analyses (*p*-values of interaction term, 0.03 for NHL and 0.02 for HL).

The analyses by lymphoma and leukemia subtype are shown in Table 5. The results were quite similar for the lymphoblastic (ALL) and the myeloblastic (AML) subtypes of AL, but the associations were less clear for T-cell ALL (67 cases) than for common B-cell ALL (544 cases). With regard to lymphoma subtypes, maternal use of any pesticide during pregnancy was strongly associated with Burkitt lymphoma (OR = 2.8; 95% CI, 1.7–4.7) but not with the other NHL subtypes. In HL, the association was restricted to the mixed-cell subtype (OR = 3.8; 95% CI, 1.3–10.9). Paternal use of any pesticide was significantly associated with T-cell ALL (OR = 3.6; 95% CI, 1.2–10.0).

Stratified analyses showed that the associations between ALL and maternal household use of any pesticide during pregnancy were stable across the different age groups. ORs of 3.1 (95% CI, 1.8–5.3), 2.1 (95% CI, 1.6–2.8), 2.0 (95% CI, 1.3–3.2), and 2.7 (95% CI, 1.6–4.7) were observed with ALL for the age groups < 2 years, 2–6 years, 7–11 years, and ≥ 12 years, respectively. ORs of 3.5 (95% CI, 1.6–7.5), 2.6 (95% CI, 1.2–5.7), and 1.2 (95% CI, 0.7–2.0) were associated with AML for the age groups < 2 years, 2–6 years, and ≥ 7 years, respectively. The results were unchanged after adjustment for parental socio-professional category or educational levels, family history of cancer, breast-feeding, early infections or child care attendance, and after exclusion of the children whose parents were farmers. The associations with pesticide exposures did not change with urbanization or housing: The ORs for the association between AL and maternal home insecticide use during pregnancy were 1.8 (95% CI, 1.2–2.6), 2.2 (95% CI, 1.5–3.2), and 1.8 (95% CI, 1.4–2.4) for children living in rural, semiurban, and urban areas, respectively, and 1.9 (95% CI, 1.5–2.5) and 1.8 (95% CI, 1.4–2.4) for children living in a house and apartment, respectively. The same absence of interaction was observed for the paternal use of pesticides.

The sensitivity analyses showed that the results were unchanged when missing values for maternal use were considered either “never used” [for example, the OR for the association between AL and maternal pesticide use was then equal to 2.1 (95% CI, 1.7–2.5)] or “ever used” [for example, the OR for the association between AL and maternal pesticide use was then equal to 2.2 (95% CI, 1.9–2.7)]. In the extreme situation where the “not known” answers were considered “never used” for cases and “ever used” for controls, a significant association between maternal insecticide use and AL (OR = 1.8; 95% CI, 1.5–2.1) or NHL (OR = 1.5; 95% CI, 1.1–2.1) was still observed.

## Discussion

The study mainly showed that both maternal and paternal household use of pesticides were significantly associated with childhood AL and NHL, but not with HL. Maternal household insecticide use during pregnancy was significantly associated with both the ALL and AML types of AL but only, for lymphomas, with Burkitt lymphoma and mixed-cell HL. The associations with herbicide and fungicide use during pregnancy were less marked.

The size of the study provided sufficient statistical power for most of the associations under study. For HL, the smallest case group, the statistical power of the study to show ORs of 1.5 and 2.0 was equal to 56% and 95%, respectively, for exposure prevalence of 40% in controls. The study may have suffered from a lack of power for that disease subgroup, but significant results were nonetheless obtained for some subtypes of the disease.

The cases were identified through the data collection system of the French National Registry of Childhood Blood Malignancies which has a high degree of exhaustiveness (> 99% of AL diagnosed in mainland France) (Clavel et al. 2004), making case selection at the identification stage unlikely. The case mothers’ participation rate was very high, about 90%. Noninclusion was attributed mainly to the child’s poor condition. However, there is no obvious reason for household pesticide exposure being related to the severity of the disease or short-term survival, particularly because the associations were similar for rural, semiurban, and urban places of residence, where health care may differ.

We randomly selected the controls from the overall population. The national telephone directory was used as the basis for random selection. Unlisted numbers were randomly generated to prevent selection of controls on socioeconomic category or related factors that might influence inclusion in the telephone directory. The quota sampling process successfully ensured that responding controls had the same distribution as the whole case group with regard to sex and age, and the same distribution as the overall population with regard to birth order and region, as shown by comparison with the French national perinatal surveys (Blondel et al. 1997, 2001, 2006). Degree of urbanization was not available in these surveys. The refusals to take part could have been related to parental socioeconomic status or educational level, which appeared higher among controls than among HL cases and, to a lesser extent, AL cases. Maternal educational level was very similar in the control group and in the French population, but paternal educational level was higher in the control group (Blondel et al. 1997, 2001, 2006). However, parental socioeconomic status and educational level were not associated with household use of pesticide in the control group. Moreover, the results were unchanged after adjustment for those variables. Pesticide use was more frequent among controls who lived in a house, and controls lived slightly more often in a house than did cases; but the estimates were similar when stratified by housing category, and adjustment for the type of housing did not modify the results. There was therefore no indication that the controls had been selected particularly on factors related to household pesticide exposure. A previous French hospital-based case–control study found lower rates of parental pesticide use among controls, about 20%, but the study was conducted in more urban areas (Menegaux et al. 2006).

The case and control interviews were conducted in the same manner, by the same interviewers, using closed questions. Misclassifications are likely to have occurred because the exposures to pesticides were described retrospectively and reported by maternal interviews. Difficulty in recalling the exposures that occurred during pregnancy probably increases with age. Recall should therefore be greater for mothers of children < 2 years of age. Interestingly, the associations with AL were slightly more pronounced for that age group, in which misclassifications might be weaker, than for the older age groups. In addition, the questionnaire did not include details on frequency of use, chemicals used, or conditions of use, which might have specified the exposure but might also have been more difficult to recall. Paternal exposure was particularly subject to imprecision and inaccuracies, because it was collected from the mother. Some sources of exposures during childhood such as maternal use of pesticide after birth or use of pediculosis lotions were not elicited. It is therefore possible that some indirect childhood exposures may not have been detected, inducing additional misclassifications. Differential recall of past pesticide exposure by cases and controls cannot be excluded, because the general public is becoming increasingly aware of the potential toxicity and carcinogenicity of pesticides. However, it is difficult to evaluate the direction of the resulting bias, if it exists, because cases could be expected to have over- or under-reported the exposure depending on whether recognition of past exposure or guilt about that exposure took precedence. Moreover, the possibility of case mothers reporting their pesticide exposure more accurately than control mothers cannot be excluded. Thus, we were unable to formulate the hypothetical sensitivity and specificity values for maternal reporting of pesticide use among cases and controls that should be required for further sensitivity analyses (Rothman and Greenland 1998; Trivers et al. 2006). However, in this study, positive associations were observed mainly for AL and NHL and mainly for maternal insecticide use during pregnancy. If systematic overreporting by case mothers explained the results, overreporting would not be expected to depend on the disease.

“Not known” answers were considered informative data, because three response options were consistently proposed: “ever used,” “never used,” and “not known.” Thus, mothers who were not sure about use of a pesticide were expected to have answered “not known,” which should have limited potential misclassifications. Moreover, the results were supported by the sensitivity analysis, which showed persistent associations even when missing data were allocated the “never used” response for cases and the “ever used” response for controls.

Only a few known factors may have confounded the results. Degree of urbanization and type of housing were independently associated with household use of pesticides in the study, with more frequent use by the controls living in a house or in a rural area; but the results were very similar with or without adjustment for those factors considered at the time of conception or interview. The results remained unchanged after adjustment for family history of cancer, birth order, early infections, and breast-feeding. Each household pesticide use might be a confounder for the others. The strong correlations made it difficult to disentangle the various pesticide exposures. The use of combined variables and multivariate analyses suggested that paternal use of pesticide was likely to be confounded by maternal use. Because the study focused on intrauterine exposure due to maternal pesticide use, the role of exposure during childhood could not be elucidated. However, the fact that the association was observed for children < 2 years of age and did not get stronger for older children points to a greater role of exposures during pregnancy. Finally, farming and occupational exposures to pesticides were very uncommon in the study population and were not related to hematopoietic cancers. Occupational exposures were therefore not confounders. Moreover, excluding children whose parents worked in agriculture or whose mothers were occupationally exposed to pesticide did not change the results.

Two exhaustive reviews on childhood cancer and pesticides have been published (Daniels et al. 1997; Zahm and Ward 1998). And four updates have been published (Infante-Rivard and Scott Weichenthal 2007; Jurewicz and Hanke 2006; Nasterlack 2006, 2007). To date, some 30 epidemiologic studies have investigated the role of pesticide exposure in childhood acute leukemia and about ten in childhood lymphoma.

Most of the studies have, however, focused on parental occupational exposure. The present study did not demonstrate any positive association with maternal occupational exposure during pregnancy, but very few mothers were involved. A positive association between parental occupational exposure to pesticides or farming and childhood AL or lymphoma has been reported in some studies (Daniels et al. 1997; Infante-Rivard and Scott Weichenthal 2007; Jurewicz and Hanke 2006; Nasterlack 2006; Zahm and Ward 1998).

Fewer studies have addressed household exposure. With regard to maternal household exposure to pesticides during pregnancy, five of the seven published studies on childhood AL (Buckley et al. 1989; Infante-Rivard et al. 1999; Leiss and Savitz 1995; Lowengart et al. 1987; Ma et al. 2002; Meinert et al. 2000; Menegaux et al. 2006) reported significant and positive ORs. Lowengart et al. (1987) reported significant ORs of 3.2 and 9.0, respectively, for indoor and outdoor maternal use of pesticide during pregnancy or nursing. Infante-Rivard et al. (1999) observed significant associations between ALL and parental use of indoor insecticides during pregnancy, indoor plant insecticides (OR = 2.0; 95% CI, 1.3–2.9), outdoor herbicides (OR = 1.8; 95% CI, 1.3–2.6), and products for trees (OR = 1.7; 95% CI, 1.1–2.6). They reported dose–response relationships with some outdoor pesticide uses. Menegaux et al. (2006) reported ORs of 1.8 (95% CI, 1.2–2.8) and 2.5 (95% CI, 0.8–7.2) for maternal use of home insecticides and garden pesticides during pregnancy. Leiss and Savitz (1995) reported ORs of 3.0 (95% CI, 1.6–5.7) and 1.1 (95% CI, 0.6–1.9) for parental hanging of pest strips in the home and parental garden treatment. Ma et al. (2002) found positive dose–response relationships and reported ORs of 2.1 (95% CI, 1.3–3.5), 0.8 (95% CI, 0.4–1.4), and 1.6 (95% CI, 0.9–3.0) for AL and insecticides use, flea control product use, and herbicide use, respectively. In a study of AML, Buckley et al. (1989) did not distinguish between indoor and outdoor maternal exposure to pesticide during pregnancy, and they reported nonsignificant ORs of 1.4 for less than one use per week and 0.9 for between one and two uses per week. However, the authors reported that eight cases and no control mothers were exposed to pesticides on most days during pregnancy. Meinert et al. (2000) reported a nonsignificant OR of 1.4 for parental indoor use of insecticides. In general, it would appear that indoor pesticide use during pregnancy may be more strongly associated with AL than the products used for gardening. In two studies, gestational single exposures to pesticides applied by pest exterminators were investigated. ORs of 2.2 (95% CI, 1.0–4.8) (Ma et al. 2002) and 0.4 (95% CI, 0.1–1.2) (Leiss and Savitz 1995) were reported.

Childhood exposure due to parental household use of pesticides has been investigated in seven studies (Buckley et al. 1989; Infante-Rivard et al. 1999; Leiss and Savitz 1995; Ma et al. 2002; Meinert et al. 1996, 2000; Menegaux et al. 2006). Four of the studies reported positive and significant results (Infante-Rivard et al. 1999; Leiss and Savitz 1995; Meinert et al. 1996; Menegaux et al. 2006), three with garden products (Infante-Rivard et al. 1999; Meinert et al. 1996; Menegaux et al. 2006), and three with indoor products (Infante-Rivard et al. 1999; Leiss and Savitz 1995; Menegaux et al. 2006). Buckley et al. (1989) reported a significant dose–response gradient for AL, although the associations were not statistically significant. Two studies investigated the use of pesticides for pets during pregnancy or childhood and did not find any positive association (Infante-Rivard et al. 1999; Ma et al. 2002). Menegaux et al. (2006) reported a significant OR of 1.9 (95% CI, 1.1–3.3) for childhood AL and the use of pediculosis lotion during childhood.

Only three studies have investigated the relationship between household exposure to pesticides and childhood lymphoma (Buckley et al. 2000; Leiss and Savitz 1995; Meinert et al. 2000). Two studies addressed only NHL (Buckley et al. 2000; Meinert et al. 2000). The third investigated both NHL and HL (Leiss and Savitz 1995). In all three studies, at least one household pesticide exposure was significantly associated with childhood lymphoma. Leiss and Savitz (1995) reported an increased risk of lymphoma with specialist pest extermination exposure during early childhood (OR = 1.8; 95% CI, 1.1–2.9]). However, for lymphoma, they did not report the strong association with pest-strip exposure that they observed with AL. Meinert et al. (2000) observed positive and significant ORs for NHL and maternal exposure to indoor pesticide use during pregnancy (OR = 3.7; 95% CI, 1.8–7.6) and specialist pest extermination exposure during pregnancy or childhood (OR = 2.6; 95% CI, 1.2–5.7), whereas for AL the latter OR was 1.3 (95% CI, 0.8–2.3). Meinert et al. (2000) also found a significant dose–response trend with parental indoor insecticide use during childhood. Buckley et al. (2000) reported ORs consistently greater than unity and a nearly significant (*p* = 0.05) trend for maternal exposure to insecticides during pregnancy, and significant ORs for childhood exposure to pesticides (OR = 2.4; 95% CI, 1.4–4.0) and professional pest extermination around the home during pregnancy (OR = 3.0; 95% CI, 1.4–6.2).

In the present study, the associations were stronger for common B-cell ALL and AML than for T-cell ALL or mature B-cell ALL. Burkitt lymphoma was also more strongly associated with maternal pesticide use during pregnancy than the other NHL. With regard to HL, only the mixed-cell subtype was associated with exposure. It is noteworthy that the two types of lymphoma associated with maternal pesticide use during pregnancy are both Epstein-Barr virus–related lymphomas, which may suggest that some kind of interaction between pesticide exposure and susceptibility to viral lymphomagenesis might exist. Few other studies have investigated the association between childhood leukemia or lymphoma subtypes and household pesticide use. Relatively similar associations with ALL and acute non-lymphoblastic leukemia were reported in each study (Lowengart et al. 1987; Ma et al. 2002; Menegaux et al. 2006). Buckley et al. (2000) found quite similar positive associations for all subtypes of childhood NHL.

Overall, increased risks of childhood AL or lymphoma have been reported for children exposed to household pesticides. Intrauterine pesticide exposure seems to have been a little more consistently associated with childhood leukemia and lymphoma than childhood exposure.

In this study, as in all the published studies, differential misclassification bias cannot be ruled out. Obtaining accurate prospective exposure assessments thus remains crucial.

## Conclusion

In conclusion, the study findings strengthen the hypothesis that domestic use of pesticides may play a role in the etiology of childhood hematopoietic malignancies and support the view that the prenatal period may be a particularly vulnerable time window. The consistency of the findings with those of previous studies on AL in different populations, at different time periods, and using different designs, raises the question of the advisability of preventing pesticide use by pregnant women, even though a causal relationship still must be more fully documented.

## Figures and Tables

**Table 1:** Distribution of cases and controls by the stratification variable, age × sex (16 categories) used for quota sampling.

			AL				
Age (year)	Controls [*n* = 1,681 (%)]	All study cases^a^ [*n* = 1,460 (%)]	All [*n* = 764 (%)]	ALL (*n* = 646)	AML (*n* = 100)	HL [*n* = 130 (%)]	NHL [*n* = 166 (%)]
Boys							
0–1	201 (12)	125 (9)	53 (7)	34	17	0 (0)	1 (1)
2	79 (5)	83 (6)	54 (7)	49	5	0 (0)	3 (2)
3	87 (5)	84 (6)	59 (8)	56	2	1 (1)	9 (6)
4	89 (5)	78 (5)	48 (6)	42	4	0 (0)	11 (7)
5–6	126 (8)	126 (9)	59 (8)	49	6	7 (5)	26 (16)
7–8	96 (6)	85 (6)	35 (5)	29	5	11 (8)	18 (11)
9–11	137 (8)	127 (9)	59 (8)	49	9	22 (17)	24 (14)
12–14	117 (7)	108 (7)	47 (6)	37	6	21 (16)	26 (16)
Girls							
0–1	168 (10)	133 (9)	60 (8)	42	18	0 (0)	0 (0)
2	74 (4)	70 (5)	49 (6)	46	3	0 (0)	2 (1)
3	79 (5)	70 (5)	50 (7)	47	2	0 (0)	5 (3)
4	56 (3)	61 (4)	38 (5)	35	3	1 (1)	5 (3)
5–6	102 (6)	84 (6)	60 (8)	55	5	1 (1)	7 (4)
7–8	67 (4)	65 (4)	40 (5)	34	5	2 (2)	8 (5)
9–11	88 (5)	63 (4)	34 (4)	26	7	10 (8)	8 (5)
12–14	115 (7)	98 (7)	19 (2)	16	3	54 (42)	13 (8)

a Includes leukemia, lymphoma, neuroblastoma, and brain tumor.

**Table 2:** Socioeconomic and familial characteristics of cases and controls.

	AL			HL			NHL		
	Cases (*n* = 764)	Controls (*n* = 1,681)	OR^a^ (95% CI)	Cases (*n* = 128)	Controls (*n* = 848)	OR^a^ (95% CI)	Cases (*n* = 165)	Controls (*n* = 1,312)	OR^a^ (95% CI)
Maternal educational level									
≤High school	465	979	1.0	91	532	1.0	97	795	1.0
> High school	299	701	0.9 (0.8–1.1)	33	315	0.7 (0.4–1.0)	67	516	1.1 (0.8–1.6)
Paternal educational level									
≤High school	526	1,063	1.0	102	569	1.0	112	851	1.0
> High school	230	601	0.8 (0.6–0.9)	22	270	0.5 (0.3–0.8)	51	445	1.0 (0.7–1.4)
Parental professional category at interview^b^									
Intellectual/scientific jobs, managers, and intermediate professions	279	715	1.0	38	360	1.0	67	557	1.0
Administrative and sales workers	224	477	1.2 (1.0–1.5)	33	221	1.5 (0.9–2.5)	41	356	1.0 (0.6–1.5)
Service workers	96	215	1.2 (0.9–1.5)	29	133	1.9 (1.1–3.3)	32	179	1.3 (0.8–2.0)
Factory and agricultural workers, unemployed	165	274	1.5 (1.2–1.9)	28	134	2.1 (1.2–3.7)	25	220	0.9 (0.6–1.6)
Place of residence at the time of diagnosis									
Rural	250	601	1.0	44	294	1.0	44	459	1.0
Semiurban	183	391	1.1 (0.9–1.4)	34	200	1.1 (0.7–1.8)	52	305	1.8 (1.2–2.8)
Urban	329	689	1.1 (0.9–1.4)	50	354	1.0 (0.6–1.6)	69	548	1.3 (0.9–2.0)
Housing after birth									
Only flat	199	395	1.0	25	147	1.0	35	265	1.0
At least once in a house	550	1,255	0.9 (0.7–1.1)	99	682	0.7 (0.4–1.1)	129	1,018	0.9 (0.6–1.3)
At least once in a farm	15	31	0.9 (0.5–1.7)	4	19	1.0 (0.3–3.4)	1	29	0.2 (0.1–1.9)
Housing during pregnancy									
Only flat	368	709	1.0	64	371	1.0	78	561	1.0
At least once in a house	387	948	0.8 (0.7–0.9)	62	464	0.8 (0.5–1.1)	85	729	0.8 (0.6–1.2)
At least once in a farm	9	24	0.7 (0.3–1.4)	2	13	0.9 (0.2–4.1)	2	22	0.7 (0.2–3.2)
Child contacts with cats or dogs (at least once per week)									
Yes	536	1,144	1.0 (0.8–1.2)	88	603	0.8 (0.5–1.2)	115	917	1.0 (0.7–1.4)

Analyses were restricted to children > 4 years of age for HL and > 1 year of age for NHL.

a ORs and 95% CIs were estimated by unconditional regression, adjusted for stratification variable, age × sex.

b Professional category is the best job of child’s mother or father.

**Table 3:** Household pesticide use and childhood hematopoietic malignancies.

	AL			HL			NHL		
	Controls (*n* = 1,681)	Cases (*n* = 764)	OR^a^ (95% CI)	Controls (*n* = 848)	Cases (*n* = 128)	OR^a^ (95% CI)	Controls (*n* = 1,312)	Cases (*n* = 165)	OR^a^ (95% CI)
Maternal household pesticide use during pregnancy									
Pesticide use (ever vs. never)	620	401	2.2 (1.8–2.6)#	358	60	1.3 (0.9–2.0)	516	87	1.9 (1.3–2.6)#
Insecticide use (ever vs. never)	590	383	2.1 (1.7–2.5)#	340	58	1.3 (0.9–2.0)	492	83	1.8 (1.3–2.6)#
Home insecticide (ever vs. never)	521	324	1.9 (1.6–2.3)#	311	56	1.4 (1.0–2.2)	438	66	1.4 (1.0–2.0)
Pet insecticide (ever vs. never)	204	156	2.0 (1.5–2.5)#	117	20	1.3 (0.7–2.2)	167	24	1.2 (0.7–1.9)
Garden crop insecticide (ever vs. never)	48	29	1.5 (1.0–2.5)	30	3	0.5 (0.1–1.8)	36	10	2.3 (1.1–4.9)*
Herbicide use (ever vs. never)	92	53	1.5 (1.0–2.2)*	62	9	1.1 (0.5–2.4)	77	14	1.5 (0.8–2.7)
Fungicide use (ever vs. never)	41	17	0.9 (0.5–1.7)	24	6	1.9 (0.7–5.3)	32	4	1.0 (0.3–2.9)
Paternal household pesticide use since conception									
Pesticide use (ever vs. never)	942	473	1.5 (1.2–1.8)#	508	75	1.0 (0.6–1.5)	757	108	1.7 (1.2–2.6)**
Insecticide use (ever vs. never)	732	389	1.4 (1.2–1.7)#	408	56	0.9 (0.6–1.4)	590	88	1.5 (1.0–2.1)*
Home insecticide (ever vs. never)	530	304	1.5 (1.3–1.8)#	310	46	1.1 (0.7–1.7)	437	70	1.5 (1.1–2.1)*
Pet insecticide (ever vs. never)	323	175	1.3 (1.0–1.6)*	167	29	1.2 (0.8–2.0)	259	35	1.2 (0.8–1.8)
Garden crop insecticide (ever vs. never)	245	103	1.0 (0.7–1.3)	137	17	0.9 (0.5–1.5)	191	29	1.3 (0.8–2.0)
Herbicide use (ever vs. never)	685	318	1.2 (1.0–1.4)	374	55	1.1 (0.7–1.6)	552	79	1.5 (1.0–2.2)*
Fungicide use (ever vs. never)	271	126	1.1 (0.9–1.4)	156	28	1.3 (0.8–2.2)	223	35	1.5 (0.9–2.2)
Parental agricultural occupation during pregnancy									
Mother (ever vs. never)	28	3	0.2 (0.1–0.8)*	15	4	3.2 (0.9–11.8)	23	0	—
Father (ever vs. never)	69	20	0.6 (0.4–1.1)	33	3	0.6 (0.1–3.1)	53	8	1.5 (0.6–3.6)
Maternal occupational contact with pesticide during pregnancy									
Any contact (vs. none)	42	21	1.2 (0.7–2.0)	29	3	0.7 (0.2–2.6)	37	5	1.1 (0.4–2.8)

Analyses were restricted to children > 4 years of age for HL and > 1 year of age for NHL.

a ORs and 95% CIs were estimated by unconditional regression, adjusted for stratification variables, age × sex, degree of urbanization, type of housing, and, for acute leukemia, birth order.

* 10^−2^ ≤ *p* <0.05.

** 10^−3^ ≤ *p* <10^−2^.

# *p* < 10^−3^.

**Table 4:** Variables combining the various types of household pesticide use.

	AL			HL			NHL		
	Controls (*n* = 1,681)	Cases (*n* = 764)	OR^a^ (95% CI)	Controls (*n* = 848)	Cases (*n* = 128)	OR^a^ (95% CI)	Controls (*n* = 1,312)	Cases (*n* = 165)	OR^a^ (95% CI)
Parental use of any pesticide									
No pesticide use by either parent	560	171	1.0	253	28	1.0	413	28	1.0
Maternal pesticide use only	101	75	2.7 (1.9–3.8)#	41	15	3.4 (1.6–7.3)**	75	16	3.3 (1.7–6.5)#
Paternal pesticide use only	418	142	1.3 (1.0–1.8)*	192	27	1.2 (0.7–2.2)	315	34	1.8 (1.1–3.1)*
Maternal and paternal pesticide use	501	313	2.5 (2.0–3.2)#	302	43	1.2 (0.7–2.1)	424	68	2.5 (1.6–4.1)#
Parental use of insecticides, herbicides, and fungicides									
No pesticide use by either parent	560	171	1.0	253	28	1.0	413	28	1.0
Maternal insecticide use only with no paternal pesticide use	84	61	2.5 (1.7–3.7)#	33	12	3.9 (1.7–9.2)**	62	14	3.6 (1.7–7.3)#
Maternal herbicide use only with no paternal pesticide use	5	4	5.0 (1.3–19)*	3	0	—	4	0	—
Maternal fungicide use only with no paternal pesticide use	2	0	—	1	0	—	1	1	12.5 (0.6–243)
At least two maternal uses with no paternal pesticide use	8	6	2.7 (0.9–8.4)	3	3	6.1 (1.1–35)*	6	1	2.3 (0.3–21.2)
Paternal insecticide use only with no maternal pesticide use	73	22	1.1 (0.6–1.8)	31	3	0.9 (0.2–3.1)	52	4	1.2 (0.4–3.5)
Paternal herbicide use only with no maternal pesticide use	94	35	1.6 (1.0–2.4)	41	6	1.2 (0.4–3.2)	68	7	1.6 (0.7–4.0)
Paternal fungicide use only with no maternal pesticide use	5	2	1.2 (0.2–6.5)	2	0	—	5	0	—
At least two paternal uses with no maternal pesticide use	231	76	1.3 (1.0–2.0)	108	13	1.0 (0.5–2.2)	176	20	1.8 (1.0–3.6)
Maternal and paternal pesticide use	501	313	2.5 (2.0–3.2)#	302	43	1.2 (0.7–2.1)	424	68	2.5 (1.6–4.1)#

Analyses were restricted to children > 4 years of age for HL and > 1 year of age for NHL.

a ORs and 95% CIs were estimated by unconditional regression, adjusted for stratification variables, age × sex, degree of urbanization, type of housing, and, for acute leukemia, birth order.

* 10^−2^ ≤ *p* < 0.05.

** 10^−3^ ≤ *p* < 10^−2^.

# *p* < 10^−3^.

**Table 5:** Parental household pesticide use by childhood hematopoietic malignancy subtype [OR [95% CI)].

	Maternal household use of pesticide (ever vs. never)				Paternal household use of pesticide (ever vs. never)			
	Any pesticide	Insecticides	Herbicides	Fungicides	Any pesticide	Insecticides	Herbicides	Fungicides
AL								
All ALL	2.3 (1.9–2.8)#	2.2 (1.8–2.6)#	1.7 (1.2–2.5)**	1.1 (0.6–2.0)	1.5 (1.2–1.9)#	1.5 (1.2–1.9)#	1.2 (1.0–1.5)	1.1 (0.9–1.5)
Common B-cell ALL	2.4 (2.0–3.0)#	2.3 (1.9–2.8)#	1.9 (1.3–2.9)**	1.1 (0.6–2.1)	1.6 (1.3–2.0)#	1.6 (1.3–1.9)#	1.4 (1.1–1.7)**	1.2 (0.9–1.5)
Mature B-cell ALL	0.8 (0.2–2.9)	0.6 (0.2–2.4)	1.5 (0.3–6.4)	—	0.6 (0.2–2.0)	0.6 (0.2–2.1)	0.8 (0.2–2.8)	0.4 (0–3.4)
T-cell ALL	1.5 (0.9–2.7)	1.5 (0.9–2.6)	0.5 (0.1–2.0)	1.2 (0.3–5.6)	1.3 (0.7–2.3)	1.5 (0.9–2.6)	0.7 (0.4–1.2)	1.3 (0.7–2.4)
AML	2.2 (1.4–3.3)#	2.1 (1.4–3.3)#	1.2 (0.5–2.8)	—	1.5 (0.9–2.4)	1.3 (0.8–2.0)	1.0 (0.7–1.7)	1.1 (0.6–2.0)
NHL								
Burkitt lymphoma	2.8 (1.7–4.7)#	2.7 (1.6–4.5)#	1.7 (0.7–4.0)	1.6 (0.5–5.7)	1.4 (0.8–2.4)	1.7 (1.0–2.8)	1.0 (0.6–1.8)	1.3 (0.7–2.4)
B-cell lymphoblastic	1.5 (0.8–3.0)	1.5 (0.8–2.9)	0.7 (0.2–3.0)	1.2 (0.2–9.4)	1.9 (0.9–4.2)	1.7 (0.8–3.3)	1.7 (0.8–3.6)	2.0 (0.9–4.5)
T-cell lymphoblastic	1.5 (0.7–3.3)	1.4 (0.6–3.0)	2.6 (0.7–9.0)	—	3.6 (1.2–10.0)*	1.1 (0.5–2.6)	2.8 (1.0–7.5)*	1.3 (0.5–3.7)
Anaplastic large cell	0.9 (0.4–2.3)	1.1 (0.4–2.5)	1.4 (0.3–6.0)	—	1.5 (0.5–4.1)	1.1 (0.4–2.7)	2.0 (0.7–5.7)	1.5 (0.5–4.4)
HL								
Nodular sclerosis	1.0 (0.6–1.7)	1.1 (0.6–1.9)	1.3 (0.5–3.1)	3.4 (1.1–10.2)*	0.8 (0.5–1.3)	0.6 (0.4–1.1)	0.9 (0.5–1.5)	0.9 (0.4–1.7)
Mixed-cell	3.8 (1.3–10.9)*	4.1 (1.4–11.8)**	0.8 (0.1–6.6)	2.5 (0.3–20.8)	1.0 (0.3–2.7)	1.0 (0.4–2.7)	1.3 (0.5–3.8)	1.9 (0.6–5.2)
Lymphocyte predominant	1.3 (0.5–3.8)	1.5 (0.5–4.2)	—	—	3.6 (0.8–16.8)	2.6 (0.8–8.3)	2.1 (0.7–6.6)	3.2 (1.1–9.9)*

Analyses were restricted to children > 4 years of age for HL subtypes and > 1 year of age for NHL subtypes and mature B-cell ALL.

a ORs and 95% CIs were estimated either by polytomous regression models for the ALL and AML types of leukemia, or by separate unconditional regressions for the subtypes of lymphomas or ALL. The models were all adjusted for age, sex, type of housing, degree of urbanization and, for acute leukemia subtypes, for birth order.

* 10^−2^ ≤ *p* < 0.05.

** 10^−3^ ≤ *p* < 10^−2^.

# *p* < 10^−3^.
